# Supplementary figures and images for: Mice Overexpressing Wild-Type RRAS2 Are a Novel Model for Preclinical Testing of Anti-Chronic Lymphocytic Leukemia Therapies
Source: Cancers (Basel). 2023 Dec 12;15(24):5817. doi: 10.3390/cancers15245817 (PMC10742337; doi:10.3390/cancers15245817)

$\alpha$  HA(rb)  
scanned

H

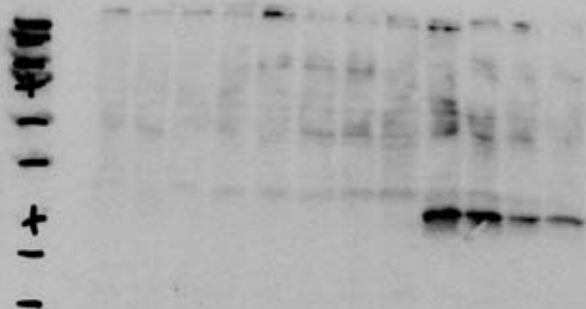

II

TC21

23vol.6

TC21

+++

++

++

+

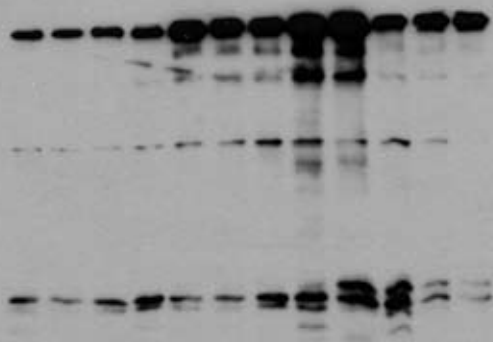

Pan Ras II

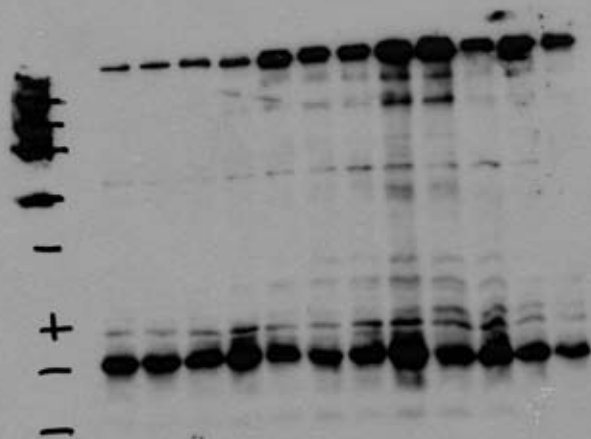

Monlon

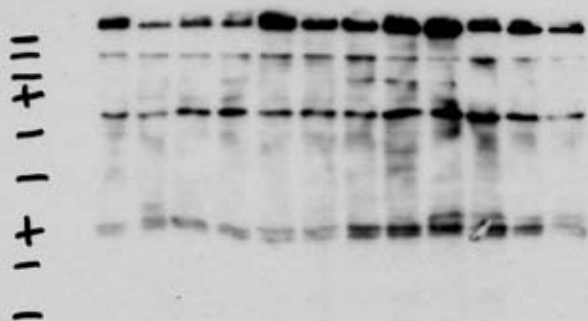

Supplement: Supplementary file 1 [file cancers-15-05817-s001.zip › cancers-2646574-supplementary.pdf]
